
JOURNAL INFORMATION
==============================
NLM Title Abbreviation: Inter Econ
Journal ID: Inter Econ
NLM Journal ID: 101086399

Inter Economics

ISSN: 0020-5346

ARTICLE INFORMATION
==============================
PMCID: PMC7276104
PMID: 32536705
DOI: 10.1007/s10272-020-0883-3
Article ID: 883
Article version: 1
Subjects: Editorial

Lessons From the Coronavirus Crisis for European Integration

Bongardt Annette abongardt@uevora.pt
1
Torres Francisco ftorres@ucp.pt
2
1 grid.8389.a 0000 0000 9310 6111 Palácio do Vimioso, CICP - University of Évora, Largo Marquês de Marialva 8, AP 94, 7002-554 Évora, Portugal
2 grid.7831.d 000000010410653X Católica Lisbon School of Business and Economics, Palma de Cima, 1649-023 Lisboa, Portugal
Electronic publication date: 2020 Jun 7
Print publication date: 2020
Volume: 55
Issue: 3
First page: 130
Last page: 131
Copyright: © The Author(s) 2020
License: Open Access This article is licensed under a Creative Commons Attribution 4.0 International License, which permits use, sharing, adaptation, distribution and reproduction in any medium or format, as long as you give appropriate credit to the original author(s) and the source, provide a link to the Creative Commons licence, and indicate if changes were made.
The images or other third party material in this article are included in the article’s Creative Commons licence, unless indicated otherwise in a credit line to the material. If material is not included in the article’s Creative Commons licence and your intended use is not permitted by statutory regulation or exceeds the permitted use, you will need to obtain permission directly from the copyright holder.
To view a copy of this licence, visit http://creativecommons.org/licenses/by/4.0/.
License URL: https://creativecommons.org/licenses/by/4.0/

issue-copyright-statement: © The Author(s) 2020

==============================

The COVID-19 pandemic caused an exogenous shock that tests the resilience of European integration. The European Union finds itself at a crossroads. National uncoordinated responses threaten core European institutions, yet the crisis is also an opportunity to advance integration and reinforce EU objectives. This calls for addressing public health governance failures, dealing with the economic and institutional fallout, protecting common institutions, building up new ones and using the Green Deal as a crisis exit strategy. The challenge comes at a time when the UK had all but monopolised the EU's attention and scarce resources with Brexit. This came at a high cost, as the Union was distracted from addressing common problems and neglected governance building.

How did we arrive at the current situation? The world did not heed warnings (e.g. of the Global Preparedness Monitoring Board report of September 2019) to think long term, prepare for a respiratory disease and cooperate on a global level for a coronavirus-type pandemic despite the many admonitions in this millennium alone (SARS, avian flu, swine flu, Ebola and Zika). In Europe, the pandemic crisis laid bare governance failures (lack of preparedness, slow responses). The European Centre for Disease Prevention and Control, created post-SARS in 2005 to assess and monitor diseases and coordinate national responses, underestimated the risk to the European population and all but disappeared from the public sphere. Although there are limits to EU public health competences (for supportive action and coordination, for proposing legally binding measures to cross-border threats on health), the Union has a broad margin of discretion on public health matters.

The coronavirus outbreak in Wuhan had been widely televised, showing overstretched hospitals and high fatality rates and the drastic measures taken by China (lockdown, cancellation of the New Year festivities), but was met with complacency in Europe. Initial scattered outbreaks in Europe were said to be controllable and did not spark preventive measures (e.g. control of all flights into Europe). The chief preoccupation was to avoid alarming the population rather than to control the spread by informing the public about the perils of not adopting social distancing behaviour. The performance of (much poorer) Asian and African countries, which did significantly better in containing the pandemic, makes governance failures even more striking.

The European Commission attempted to coordinate member states' responses, aiming to find out about preparedness and promoting joint procurement of medical supplies and equipment. Yet, like member states, it did not grasp the scale of the challenge until Italy requested the activation of the EU's Civil Protection Mechanism of European solidarity in disasters and not a single member state came forward (instead, prioritising protecting national stocks, as little or nothing had been done to produce or import protective equipment). That said, member states such as Greece that acted earlier than others upon witnessing Italy's plight, fared better.

Having failed in terms of prevention, member states have since come to converge, in a non-coordinated fashion, by adopting a variety of confinement and containment strategies in an attempt to control exponentially rising infection numbers that risk outstripping healthcare capacities and reducing them to a level that would allow for following the World Health Organization's guidance of testing, tracing and isolating. There are limits to what decentralised action can achieve against a virus that respects no borders. Any action at a local, regional or national level, even if effective in terms of permitting a tailored approach to limiting contagion, is subject to spillovers. To prevent importing cases, member states cordoned off hotspots and discriminately closed internal Schengen borders. Countries can only open up if others have comparable performance and risk profiles.

The coronavirus crisis illustrated the fragility of European common goods like the single market and the Schengen agreement and even the Economic and Monetary Union (EMU) if national measures are incompatible or insufficiently coordinated. Proposed temporary remedies are also problematic. For example, although 'green corridors' proposed by the European Commission have tried to safeguard the flow of vital goods (at the level of supply chains to limit the risk of disruptions), the free movement of people was suspended (with exceptions for some professions), which has high economic costs. Relaxing state aid rules in the crisis is not compatible with a level playing field if countries with deeper pockets support and give an unfair advantage to their own firms (thereby eroding trust in the single market). Electronic contact tracing apps hinge on public acceptance, which requires trust in data protection rules and interoperability.

So what should the EU do? First, the EU should address the economic consequences of the current pandemic crisis through its eurozone framework to complete the economic side of the EMU and avoid negative spillovers to its monetary side. In fact, the immediate response to the economic impact of the crisis is largely being built on and developed within the governance structure of the eurozone through institutions put in place or envisaged at the time of the sovereign debt crisis: European Stability Mechanism, ECB measures, budgetary rules, the still incomplete banking union and the unemployment reinsurance scheme SURE. The proposed recovery fund is a tentative step in the right direction towards common bond issuance, which would also contribute to the EMU's sustainability. The EU budget should not merely be enlarged but should provide European public goods and objectives financed by own resources.

Second, although it is a significant governance challenge, the EU should defend its interests and its own project rather than wasting time with Brexit and aligning with one side of the internal debate in the UK. Remainers favoured a different, much-diluted Union, essentially a free trade zone with some cooperation in domains where the UK wants to pursue its own objectives at the global level (trade, defence, intelligence, etc.), making use of EU infrastructure, explicitly stated in the 2016 'new settlement for the UK within Europe'. Rather than insisting on self-harming extensions, the EU should make sure that the transition period ends by 31 December 2020 and that any future agreement – should there be one – fully respects the integrity of its internal market, its common goods and European values. This is valid for all trade agreements.

Third, the crisis has shown that the EU has got its priorities – smart, social and sustainable development – right. It brought home the importance of digital technologies and may prompt a general re-evaluation of the role of the state (welfare systems and regulation). In fact, the pandemic crisis is an acute crisis within a larger environment and climate crisis context. Health and the environment have public good characteristics, with potentially large longer-term damage costs that tend not to be factored in. Moreover, the degradation of the environment, loss of biodiversity and practices like intensive and factory farming are linked to new diseases. In the novel pandemic, decision-makers adopted science-based policymaking to an extent that unfortunately has not happened in the climate crisis. The crisis context is the right time to correct market failures and promote the shift to sustainable production and consumption patterns for the benefit of public health, the environment and sustainable development. The Green Deal already furnishes an adequate exit strategy. Calls for propping up unsustainable industries in the crisis should be resisted in order not to repeat the mistakes committed in the aftermath of the financial and sovereign debt crises.

Note: The authors would like to pay tribute to Alberto Alesina whose work inspired so many of us in the field of political economy.

Open Access funding provided by ZBW — Leibniz Information Centre for Economics.

Annette Bongardt, CICP – University of Évora; and Universidade Fernando Pessoa, Porto, Portugal.

Francisco Torres, Católica Lisbon School of Business and Economics, Portugal.

